# Altitudinal and slope gradients as key determinants of *Robinia pseudoacacia* growth

**DOI:** 10.1371/journal.pone.0354736

**Published:** 2026-08-26

**Authors:** Yirui Shao, Liyan Hou, Yuntao Li, Zhikun Zhang, Jinfeng Nie, Yanli Xiao, Zhipeng Wang, Jiang Xin, Ning Wang

**Affiliations:** 1 Shandong Provincial No.4 Institute of Geological and Mineral Survey, Weifang, China; 2 Weifang Key Laboratory of Satellite Remote Sensing Intelligent Interpretation Technology, Weifang, China; 3 Shandong Open Innovation Application Laboratory for Satellite Remote Sensing Big Data, Weifang, China; 4 College of Advanced Agriculture and Life Sciences, Weifang University, Weifang, China; Shandong Normal University, CHINA

## Abstract

*Robinia pseudoacacia* is a major plantation species in northern China, yet quantitative assessments of its growth across topographic gradients remain limited. This study investigated the growth performance of *R. pseudoacacia* in relation to landform types (plains, hills, low mountains) and key topographic factors (altitude, slope) in Weifang, Shandong Province. Field surveys were conducted across 67 pure‑stand sample plots. One‑way ANOVA revealed that trees on plains had significantly greater mean diameter at breast height (DBH) and tree height than those on hills and low mountains. Linear regression showed significant negative correlations: DBH and height decreased by approximately 0.58 cm and 0.47 m per 100 m increase in altitude, and by 0.76 cm and 0.57 m per 10° increase in slope. Altitude explained slightly more variation (R² = 10.1–10.6%) than slope (R² = 5.5–6.8%). Allometric analysis indicated stable height–DBH scaling across landforms (ANCOVA, P = 0.776), with scaling exponents (b) of 0.59 (low mountain), 0.48 (hill), and 0.68 (plain). Our results quantitatively delineate growth patterns of *R. pseudoacacia* across topographic gradients, highlighting the strong association between gentle, low‑lying terrains and superior growth. These findings provide an empirical basis for site selection in afforestation projects, suggesting a preference for plain areas to enhance plantation productivity. Future studies should incorporate soil and microclimatic data to elucidate underlying mechanisms.

## 1. Introduction

Forests constitute the primary biomes of terrestrial ecosystems, serving as the structural foundation for biodiversity and driving global biogeochemical cycles, including carbon sequestration and hydrological regulation [1,2]. As a vital component of forest resources, plantation forests are an effective means to rapidly supply timber, restore degraded ecosystems, and achieve carbon neutrality goals [3,4]. Among various species, *Robinia pseudoacacia* is one of the most important plantation tree species in northern China due to its rapid growth, strong adaptability to infertile and drought-prone environments, capacity for nitrogen fixation and soil improvement, and high economic value of its wood. It has been extensively used in vegetation restoration and timber forest establishment [5,6].

However, the growth of forest trees is determined not only by their genetic characteristics but is also profoundly influenced by their topographic environment [7]. Topographic condition is one of the most direct and fundamental factors affecting forest productivity; it governs the physiological processes and growth performance of trees through the combined effect of environmental factors such as water, heat, nutrients, and aeration [8]. In different topographic landscapes—low mountains, hills, and plains—significant differences in factors such as altitude and slope lead to the spatial redistribution of resources like light, temperature, water, and soil, consequently creating distinctly different habitats [9,10]. For instance, increasing altitude is often accompanied by decreasing temperature and a shorter growing season, while steeper slopes can exacerbate water-induced soil erosion, resulting in thinner soil layers. These factors undoubtedly exert a critical influence on the growth patterns and biomass accumulation in plants [11]. Therefore, in-depth research into the relationship between different site environments and the growth of plants is crucial for scientifically guiding afforestation practices and enhancing forest productivity.

In forestry science, the concept of Site index—typically defined as the height of dominant trees at a specific reference age—is widely regarded as the standard metric for evaluating site productivity and predicting yield [12]. While site index curves have been developed for many commercial species globally, their application to *R. pseudoacacia* plantations in the complex terrain of Central Shandong remains limited. Most existing studies in this region focus on qualitative descriptions or isolated climatic/soil factors, lacking systematic models that quantify the specific influence of topographic gradients on key growth indicators while accounting for stand structural variables. This gap makes it difficult to provide precise, quantifiable scientific guidance for site selection, as traditional qualitative assessments cannot capture the continuous numerical relationships between terrain attributes and tree growth.

Currently, while numerous studies have investigated the effects of climatic or soil factors on the growth of *R. pseudoacacia*, research systematically quantifying the influence of topographic factors (such as altitude and slope) on its key growth indicators within a specific regional scope remains relatively scarce. Most studies remain qualitative, lacking the empirical evidence that can reveal the underlying quantitative relationships [13,14]. This makes it difficult to provide specific, quantifiable scientific guidance for precise plantation site selection and silvicultural management.

Based on this, this study takes *R. pseudoacacia* plantations in Weifang of Shandong Province as the research object. Through field plot investigation and data measurement, it aims to: (1) compare the growth differences of *R. pseudoacacia* across three different landforms (low mountains, hills, and plains); (2) employ regression analysis to quantitatively elucidate the numerical relationships between two key topographic factors (altitude and slope) and the growth of DBH and tree height; (3) assess the relative importance of topographic factors on its growth. This study will deepen the understanding of the environmental adaptability of *R. pseudoacacia* and provide an empirical basis and for the scientific management and afforestation of *R. pseudoacacia* plantations in this region and under similar site conditions.

## 2. Materials and methods

### 2.1 Study site and plant materials

This study was conducted in Weifang, Shandong (42.8° N, 89.2° E). The region experiences a warm temperate semi-humid continental monsoon climate with four distinct seasons. At the regional scale, the mean annual temperature is approximately 12.7°C, and the mean annual precipitation is about 650 mm. However, these climatic variables vary with altitude, with lower temperatures and locally variable precipitation occurring at higher elevations. The area features diverse geomorphology, primarily including plains, hills, and low mountains, providing an ideal setting for investigating the growth differences of *Robinia pseudoacacia* under various site conditions. According to the Chinese national standard GB/T 38590−2020 (Classification of forest land resources) and local forestry technical regulations, the classification of landforms (low mountain, hill, and plain) in this study was not based solely on absolute altitude. Instead, a multi-factor criterion integrating altitude, slope gradient, and relative relief was applied. Specifically, plots with steeper slopes (>15°) and greater relative relief (>200 m) were classified as low mountains even when their absolute altitudes overlapped with those of hill plots. Conversely, plots with gentler slopes (≤15°) and lower relative relief were classified as hills. Plains were characterized by nearly flat terrain (slope <2°) and low elevation. This approach ensures a clear distinction between landform types and avoids ambiguity arising from overlapping altitude ranges. All sampled *R. pseudoacacia* plantations were established between 1995 and 2005 (stand age 20–30 years). Planting stock was sourced from local nurseries, with initial spacing of approximately 2 m × 3 m (≈1667 stems/ha) across all sites. Post-planting management, including weeding and thinning, followed regional forestry guidelines and was similar among the three landform types. No fertilization or irrigation was applied in any of the sampled stands. No ethical approval was required for this study as it involved only non‑destructive measurements of planted trees.

### 2.2 Data source and plot investigation

**Fig 1:**
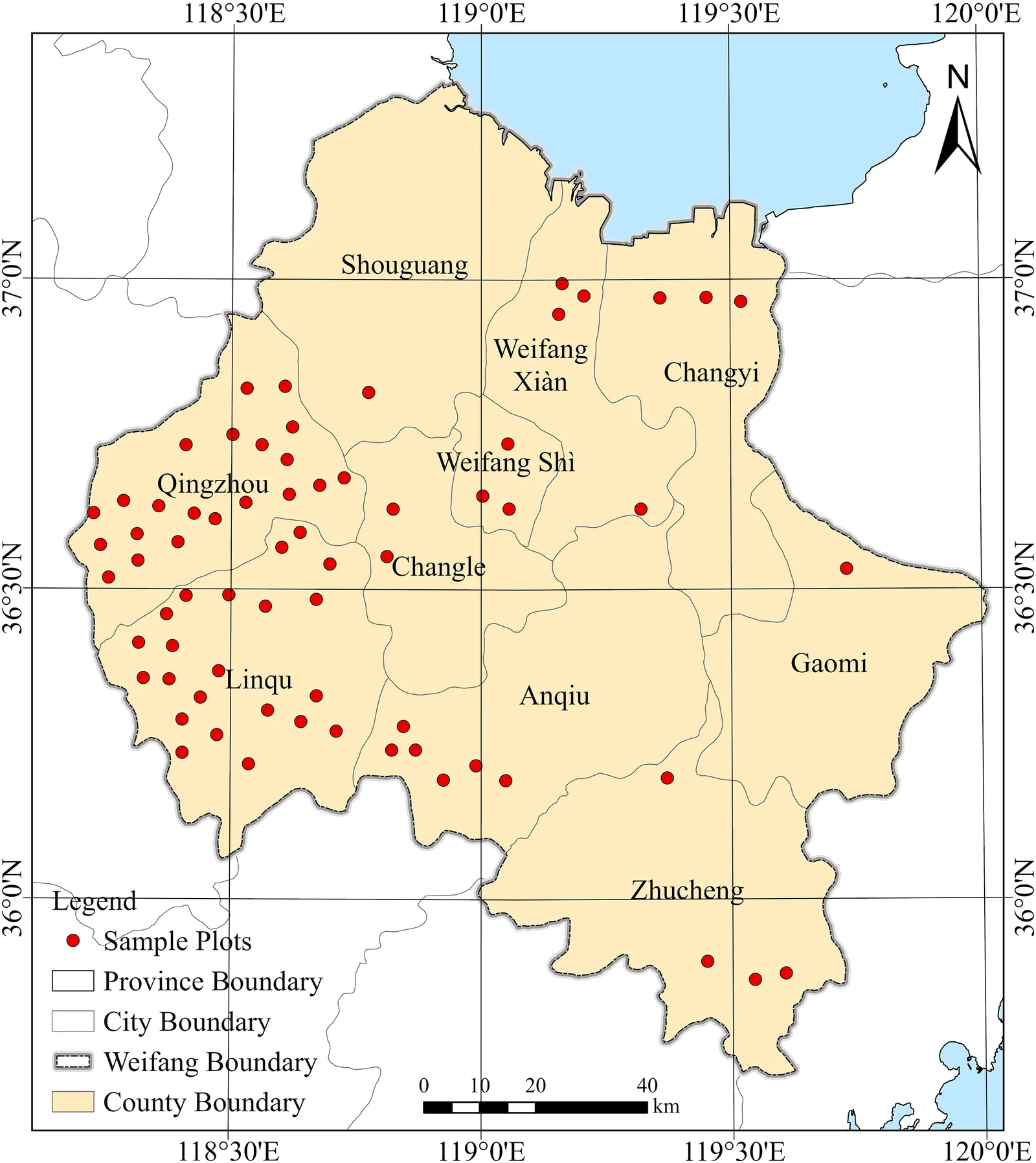
Location of the study area and distribution of sample plots. Administrative boundary data for county-level divisions were obtained from the Database of Global Administrative Areas (GADM, https://gadm.org), which permits the use of its data for creating maps for academic publication. In this dataset, the administrative unit labeled “Weifang Xian” corresponds to the current four central urban districts of Weifang City (Weicheng, Fangzi, Hanting, and Kuiwen districts). Basemap layers from Natural Earth (https://www.naturalearthdata.com) were used for additional geographic context. Study sites were added by the authors.

The data for this study were obtained from the 2024 remeasurement survey of the continuous forest inventory conducted in Weifang (Fig 1). A total of 67 representative circular sample plots were established within the study area using a systematic sampling method based on a 2 km × 2 km grid from the Weifang 2024 Forest Stock Volume Survey. Among these, 29 plots were located in low mountains, 19 in hills, and 19 in plains (see Table 1). Plots were spatially independent, with a minimum distance of at least 500 m between plot centers to avoid spatial autocorrelation. The study area encompasses all three landform types, with plots distributed across the natural range of *R. pseudoacacia* in central Shandong Province. Each plot covered an area of 0.04 ha (radius = 11.28 m). To ensure sample homogeneity and minimize confounding factors, all selected plots were pure *R. pseudoacacia* stands. Within each plot, the diameter at breast height (DBH) and total height of all living *R. pseudoacacia* trees with DBH ≥ 5 cm were measured. The average altitude of each plot was recorded using a high-precision GPS receiver, and the average slope was determined using a clinometer. Stand density (stems per hectare) for each plot was calculated based on the number of living trees with DBH ≥ 5 cm within the 0.04 ha circular plot. Mean stand density across all plots is reported in Table 1.

**Table 1:** Basic characteristics (mean ± SD, with ranges in parentheses) of the 67 sample plots across three landforms in the study area.

Variables	Plains	Hills	Low Mountains
**Number of Plots**	19	19	29
**Altitude (m)**	75.4 ± 64.4	285.6 ± 66.4	446.6 ± 156.1
**Slope (°)**	0.21 ± 0.42	14.74 ± 6.66	20.66 ± 10.64
**Stand Age (yr)**	24.5 ± 2.1	25.2 ± 2.8	23.8 ± 3.2
**Tree Density (stems/ha)**	1250 ± 320	1480 ± 410	1320 ± 380
**DBH (cm)**	13.25 ± 4.55	9.06 ± 3.57	9.94 ± 2.36
**Height (m)**	8.90 ± 4.06	5.77 ± 1.57	6.82 ± 2.45
**Canopy Closure**	0.64 ± 0.21	0.54 ± 0.25	0.49 ± 0.24

### 2.3 Statistics

Statistical analyses were performed to examine the variation in growth indicators (DBH and height) of *Robinia pseudoacacia* across the different topographic gradients. First, one-way ANOVAs followed by Duncan’s multiple comparison were used to test the diﬀerences among landform groups. To quantify the relationships between topographic factors and tree growth, simple linear regression models were developed. Separate models were fitted with plot mean DBH or mean height as the dependent variable, and either mean plot altitude or mean plot slope as the independent variable. The significance of each regression model was evaluated using an F-test. The coefficient of determination (R²) was reported as a measure of the proportion of variance in growth explained by the topographic factor. For the allometric analysis, tree‑level data (DBH and height) were available for 29 trees in low mountain plots, 19 in hill plots, and 19 in plain plots. All statistical analyses were conducted using SPSS 26.0 (IBM Corp., Armonk, NY, USA), and figures were generated with Origin 2024 (OriginLab Corp., Northampton, MA, USA).

## 3. Results

### 3.1 Variations in growth indicators under different topographic conditions

**Fig 2:**
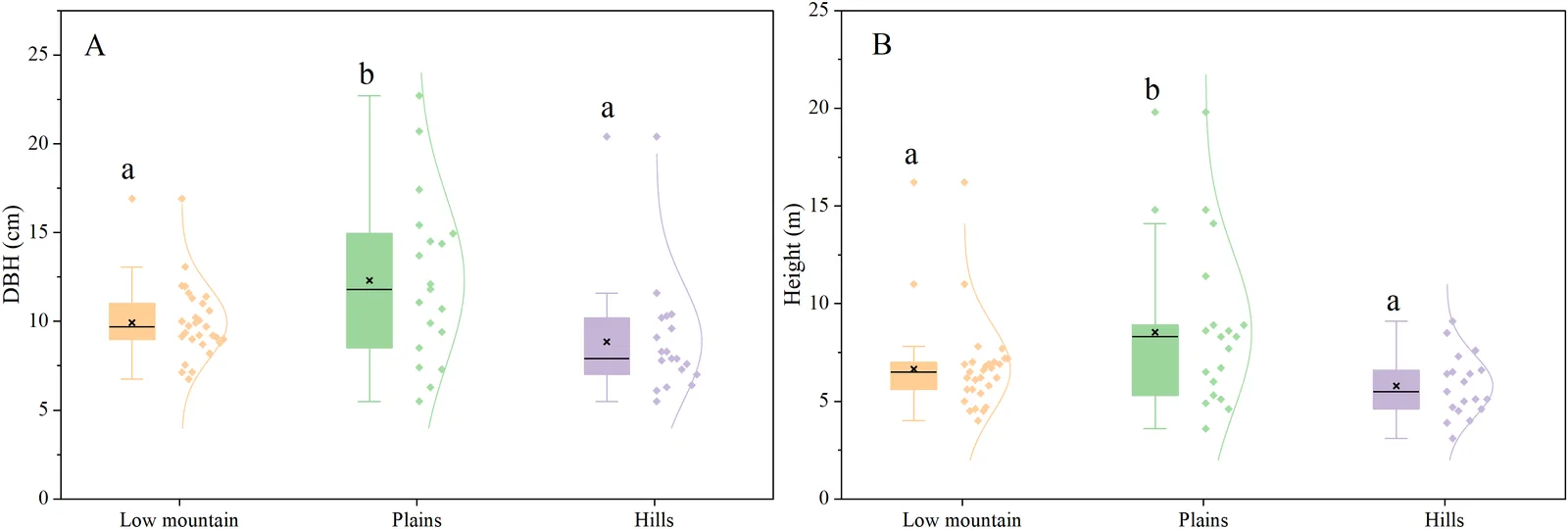
Differences in growth indicators of *R. pseudoacacia* under different topographic conditions. In the boxplots, the box represents the interquartile range (IQR), and the horizontal line within the box indicates the median. The upper and lower edges of the box correspond to the first and third quartiles, respectively. The whiskers of the boxplot extend to 1.5 times the IQR, and points beyond the whiskers are considered outliers. The scattered dots represent the raw data points, with different colors corresponding to different topographic groups. Different lowercase letters indicate significant differences among different landforms at the P < 0.05. n = 19 ~ 29.

The growth performance of *R. pseudoacacia* exhibited distinct patterns across different landforms. The results showed that for mean DBH (Fig 2A), trees on the plains exhibited the largest mean DBH, which was statistically greater than that of those in both low mountains and hills (P < 0.05). No significant difference in DBH was found between low mountain and hill sites. A similar pattern was observed for mean tree height (Fig 2B): trees on the plains achieved significantly greater height than those in low mountains and hills (P < 0.05), with no significant difference between the latter two.

### 3.2 Regression analysis of topographic features on growth indicators

**Fig 3:**
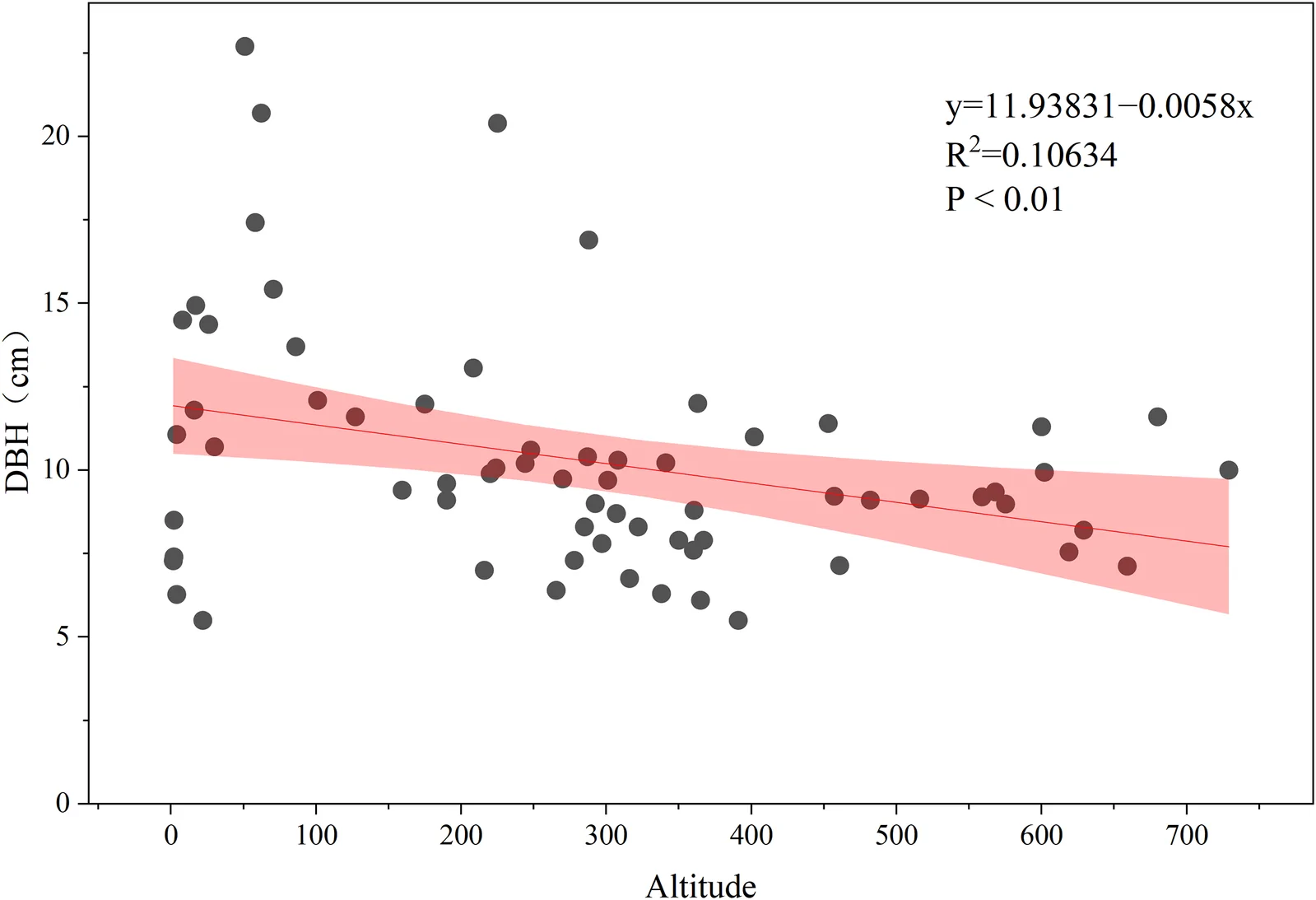
Regression relationships between altitude and DBH of *R. pseudoacacia.* Each black dot represents one sample plot (n = 67 plots). The solid red line indicates the fitted linear regression model, and the equation of the line is shown in the top right corner. The shaded pink band surrounding the regression line represents the 95% confidence interval (95% CI) of the fitted model. The coefficient of determination (R^2^) and the significance level (P-value) are also provided to indicate the goodness of fit and statistical significance of the relationship.

**Fig 4:**
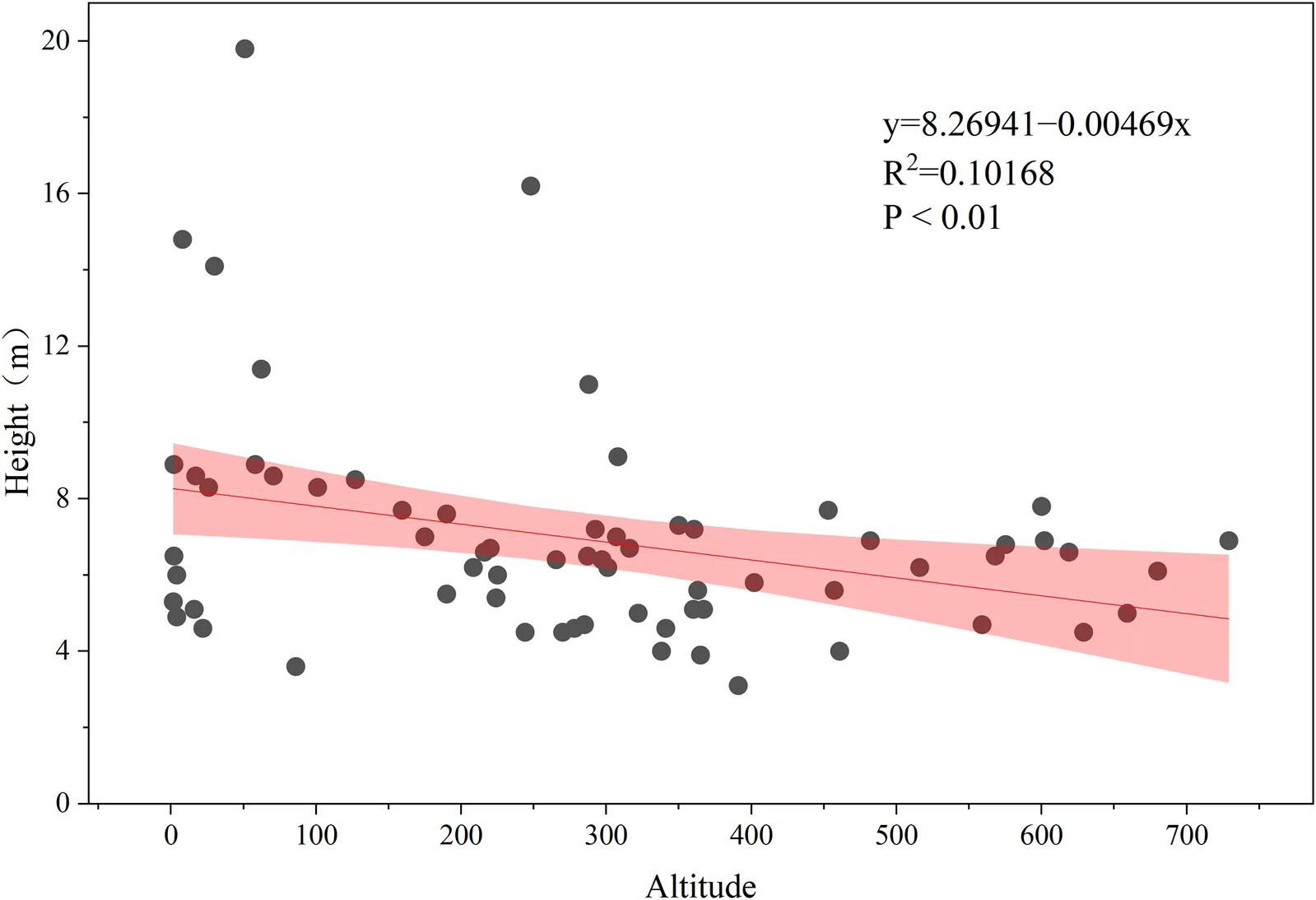
Regression relationships between altitude and height of *R. pseudoacacia.* Each black dot represents one sample plot (n = 67 plots). The solid red line indicates the fitted linear regression model, and the equation of the line is shown in the top right corner. The shaded pink band surrounding the regression line represents the 95% confidence interval (95% CI) of the fitted model. The coefficient of determination (R^2^) and the significance level (P-value) are also provided to indicate the goodness of fit and statistical significance of the relationship.

**Fig 5:**
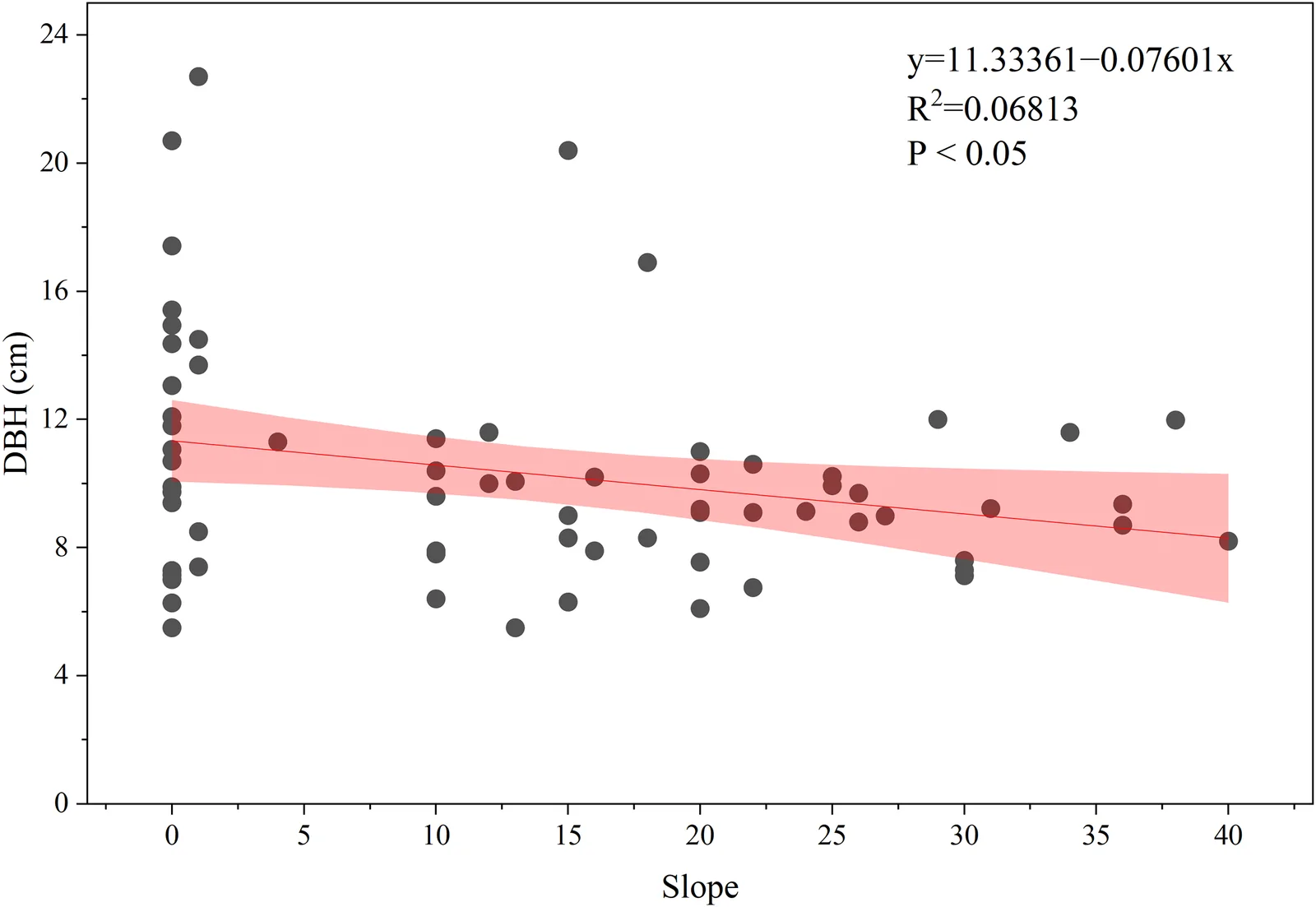
Regression relationships between slope and DBH of *R. pseudoacacia.* Each black dot represents one sample plot (n = 67 plots). The solid red line indicates the fitted linear regression model, and the equation of the line is shown in the top right corner. The shaded pink band surrounding the regression line represents the 95% confidence interval (95% CI) of the fitted model. The coefficient of determination (R^2^) and the significance level (P-value) are also provided to indicate the goodness of fit and statistical significance of the relationship.

**Fig 6:**
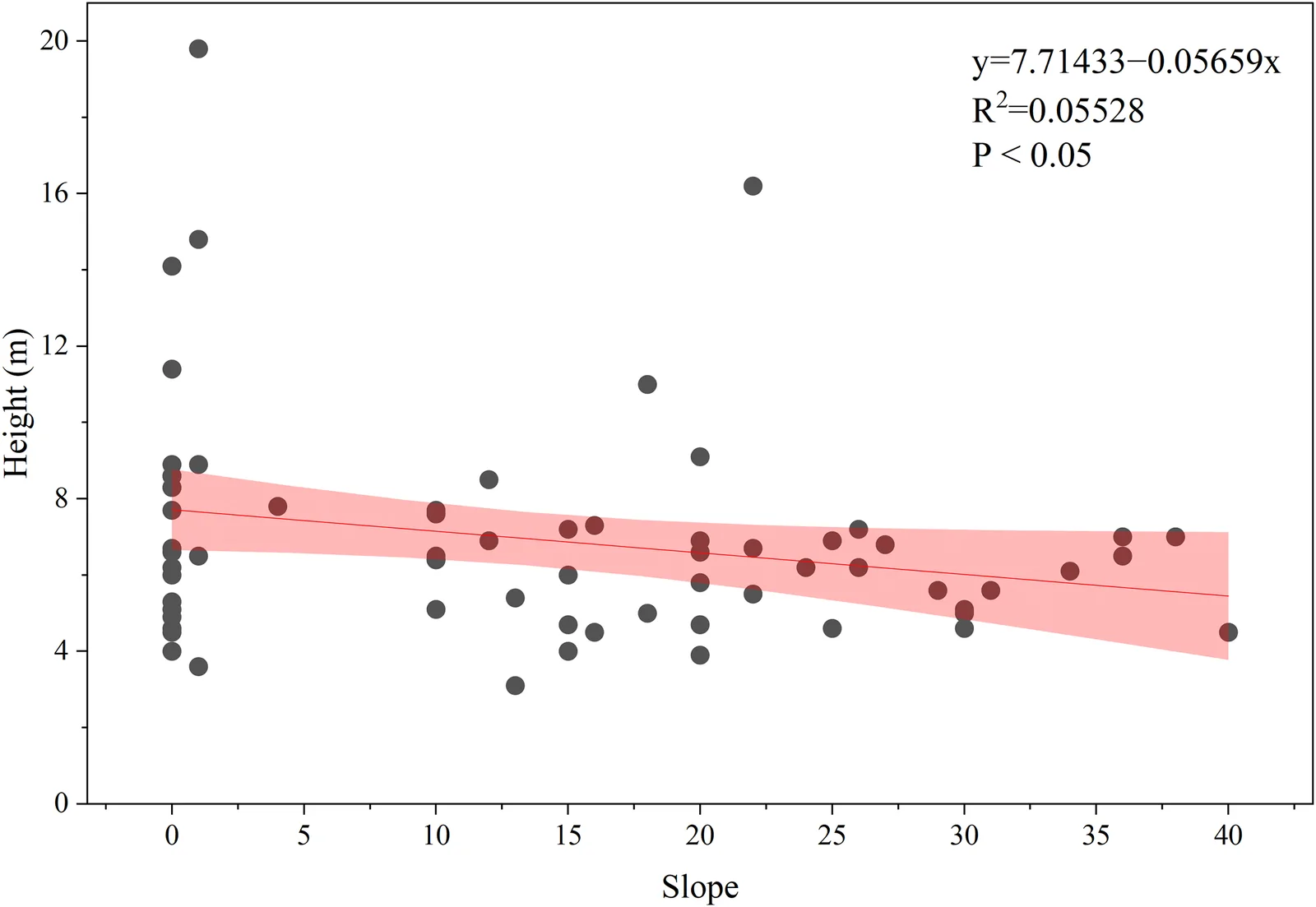
Regression relationships between slope and height of *R. pseudoacacia.* Each black dot represents one sample plot (n = 67 plots). The solid red line indicates the fitted linear regression model, and the equation of the line is shown in the top right corner. The shaded pink band surrounding the regression line represents the 95% confidence interval (95% CI) of the fitted model. The coefficient of determination (R^2^) and the significance level (P-value) are also provided to indicate the goodness of fit and statistical significance of the relationship.

To quantify the effects of topographic features on the growth of *R. pseudoacacia*, linear regression models were established between altitude, slope, and tree growth indicators. Altitude showed a statistically significant but weak negative correlation with both DBH (Fig 3: y = 11.938 − 0.0058x, R² = 0.106, P < 0.01) and height (Fig 4: y = 8.269 − 0.0047x, R² = 0.102, P < 0.01), with altitude explaining only about 10% of the variation in each growth metric. Similarly, slope showed statistically significant but weak negative correlations with mean DBH (Fig 5: y = 11.334 − 0.0760x, R² = 0.068, P < 0.05) and mean height (Fig 6: y = 7.714 − 0.0566x, R² = 0.055, P < 0.05), with slope explaining only 5–7% of the variation in each growth metric. Between the two topographic features, altitude had a slightly stronger explanatory power (higher R² values) on the growth of *R. pseudoacacia* than slope. Between the two growth indicators, DBH was more sensitive to changes in topographic features than height (higher R² values).

### 3.3 Allometric relationship between DBH and height

**Fig 7:**
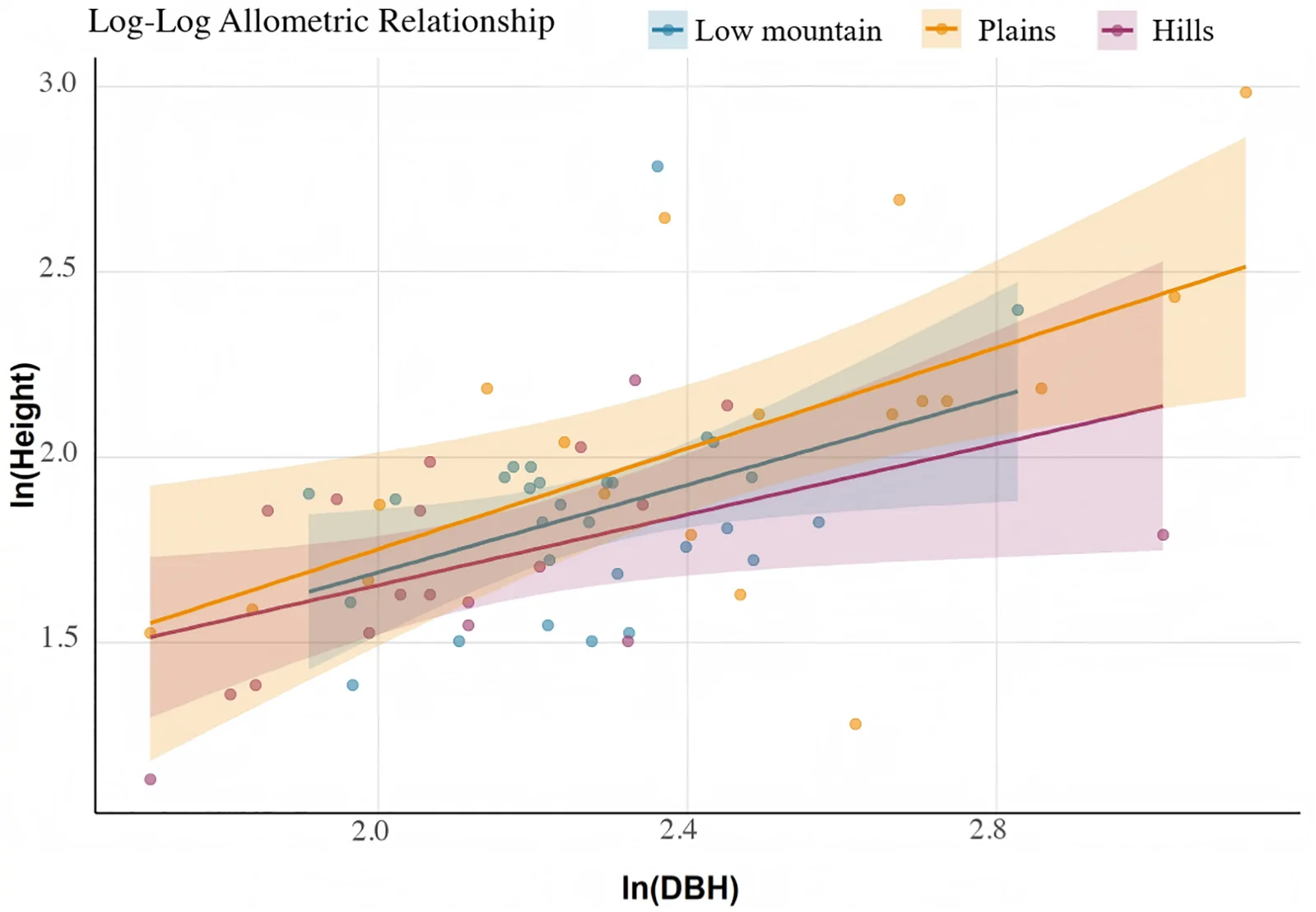
Allometric relationships between DBH and tree height of *Robinia pseudoacacia* across three landforms (low mountain, hill, plain). (A) Raw-scale scatterplot with fitted power curves (H = a·D^b). (B) Log-log transformed scatterplot with linear regression lines. Shaded bands represent 95% confidence intervals of the fitted lines. The scaling exponents (b) did not differ significantly among landforms (ANCOVA, P = 0.776). For low mountain: b = 0.59, R² = 0.17; hill: b = 0.48, R² = 0.25; plain: b = 0.68, R² = 0.38.

The allometric relationships between DBH and height were examined separately for each landform using log‑transformed linear regressions (Fig 7). For low mountain plots (n = 29 trees), the scaling exponent (b) was 0.590 ± 0.247 (mean ± SE), with an R² of 0.174 (P = 0.024). The corresponding power‑law equation was H = 1.66·D^0.59. For hill plots (n = 19), b was 0.476 ± 0.199 (R² = 0.251, P = 0.029), and the equation was H = 2.02·D^0.48. For plain plots (n = 19), b was 0.679 ± 0.213 (R² = 0.375, P = 0.005), with the equation H = 1.48·D^0.68. An ANCOVA test revealed no significant interaction between landform and ln(DBH) (F = 0.25, P = 0.776), indicating that the allometric slopes did not differ statistically among the three landform types. Thus, despite the significant differences in absolute DBH and height across landforms, the intrinsic height–diameter scaling relationship of *Robinia pseudoacacia* remained stable across the topographic gradient from low mountains to plains.

## 4. Discussion

This study quantified the growth performance of *R. pseudoacacia* across distinct topographic settings and established regression models to characterize its associations with altitude and slope. The results support the hypothesis that growth is superior on plains relative to hills and low mountains, likely due to reduced environmental stress. The following sections interpret these patterns and discuss their implications.

### 4.1 Interpretation of observed growth patterns across landforms

Consistent with the principle that forest site productivity is closely linked to topographic and edaphic conditions [12], DBH and height were significantly greater on plains than on hills or low mountains (Fig 2). This disparity is consistent with the interpretation that landform mediates the availability and redistribution of water, heat, and nutrients [15,16], although these factors were not directly measured in this study. On plains, gentle topography minimizes soil erosion and favors deposition, leading to deeper, more fertile soils with greater water- and nutrient-holding capacity [17]. This favorable edaphic environment supports extensive root development and sustained resource uptake, promoting both radial and vertical growth. Additionally, the relatively stable microclimate—with attenuated temperature fluctuations and higher plant-available water—favors continuous physiological activity and carbon assimilation [18].

In contrast, hills and low mountains present a suite of abiotic constraints. Steeper slopes accelerate soil and water erosion [19,20], stripping nutrient-rich topsoil and leaving shallow, rocky, and impoverished substrates. These conditions restrict root expansion and directly limit water and nutrient acquisition, thereby curbing biomass accumulation. Furthermore, increasing altitude is generally associated with lower temperatures, shorter growing seasons, and often higher wind exposure [21,22], although site-specific microclimatic data were not collected in this study, which collectively reduce photosynthetic efficiency, increase respiratory costs, and slow metabolic rates. The synergy between edaphic limitation and climatic stress creates a particularly challenging environment on steeper, higher-elevation sites, which aligns well with the significant negative growth–altitude and growth–slope relationships revealed by our regression models (Figs 3 and 4).

### 4.2 Quantitative relationships and their implications

This study provides a quantitative estimate of the influence of altitude and slope on growth. The regression models indicate that DBH and height decrease by approximately 0.58 cm and 0.47 m per 100 m rise in altitude, and by 0.76 cm and 0.57 m per 10° increase in slope. These relationships offer tangible parameters for preliminary yield prediction and site-suitability assessment in regional afforestation planning.

Altitude explained slightly more variation in growth (R² = 10.1–10.6%) than slope (R² = 5.5–6.8%), suggesting that hydrothermal variation linked to elevation—such as temperature decline and growing-season shortening—may be a more pervasive limiting factor in this region than erosion-related soil limitations associated with slope. It is important to note, however, that the models’ explanatory power was modest (R² < 11%), underscoring that tree growth is a multivariate process. Altitude and slope are meaningful but partial predictors; a substantial portion of growth variability likely stems from factors not measured here, including soil depth, nutrient availability, soil moisture regime, stand density, management history, and biotic interactions [23,24]. Thus, the models should be interpreted as describing important environmental correlations rather than providing a complete causal explanation.

Between the two growth traits, DBH was slightly more sensitive to topographic variation than height. This may reflect differing physiological drivers: DBH (radial growth) is strongly coupled to soil resource acquisition, whereas height growth is often prioritized under resource stress to maintain light competitiveness, potentially buffering it against short- to medium-term edaphic and climatic constraints. Although we did not develop formal site index curves due to uncertainties in stand age (caused by coppicing management and incomplete planting records), our topographic regression models serve a similar purpose by linking measurable terrain attributes to growth potential. This approach offers a practical alternative for site evaluation when precise age data are lacking, and aligns with the original intent of site index [12].

The relatively low R² values (5.5–10.6%) indicate that altitude and slope, while statistically significant, explain only a modest fraction of the variation in tree growth. This is expected given the multivariate nature of forest growth [25]. Several potentially important variables were not measured in this study. First, stand density, which can influence growth through competition, was estimated (Table 1) but its inclusion in multiple regression models did not substantially alter the significance of topographic effects. Second, soil properties (e.g., depth, organic carbon, nutrient availability) were not directly measured; they are conceptually important but were beyond the scope of this field survey. Third, microclimatic data (temperature, precipitation, growing season length) were not collected on site, although altitude serves as a well-established proxy for such factors in mountainous regions. The modest explanatory power does not invalidate the significant negative trends; rather, it underscores that future studies should integrate direct edaphic and climatic measurements to better understand the mechanisms underlying the observed topographic patterns.

### 4.3 Limitations and future research perspectives

This study provides a clear, quantitative description of *R. pseudoacacia* growth across topographic gradients, but several limitations must be acknowledged. First, we did not directly measure temperature and precipitation at each plot. We fully agree that including such data would allow a more mechanistic separation of climatic versus topographic effects. However, our study was designed to quantify the integrated, net effect of topographic gradients as they operate in actual managed landscapes, rather than to partition these drivers independently. It is worth emphasizing that altitude in this region is not merely a proxy for temperature—it also integrates changes in growing-season length, atmospheric moisture, soil development, and erosion intensity that covary with elevation. The modest R² values (5.5–10.6%) indicate that other unmeasured factors also contribute substantially to growth variation, which we explicitly acknowledge. Thus, our regression models are intended as empirical decision-support tools for site selection, rather than as causal explanatory models of tree growth. Second, direct measurements of soil properties (texture, organic carbon, nutrient pools) and site-specific microclimate (temperature, precipitation, frost duration) were not collected. Third, although stand density was estimated (Table 1), it was not included as a covariate in the main regression models because preliminary analyses showed it did not significantly alter the topographic effects. Fourth, the sample size of 67 plots, while adequate for detecting the reported patterns, limits the generalizability of the regression equations to wider landscapes. Future research should prioritize direct measurements of soil and microclimate, employ larger stratified random samples, and consider multivariate models that include biotic factors (e.g., competition, stand age). Mechanistic studies on physiological responses (e.g., photosynthesis, water use efficiency) to topographic stress are also needed. Despite these limitations, the consistent and significant trends offer an empirical foundation for site evaluation.

The allometric analysis revealed that although *R. pseudoacacia* trees on plains grew significantly larger in both DBH and height than those on hills and low mountains (Fig 2), the scaling exponent (b) relating height to DBH did not differ significantly among the three landforms (P = 0.776). This finding suggests that the intrinsic height–diameter allocation strategy of *R. pseudoacacia* is conservative and remains largely unchanged across the topographic gradient from low mountains to plains. In other words, while adverse terrain reduces overall growth rates – leading to smaller trees at a given age – it does not fundamentally alter the proportional relationship between height and diameter growth. Such allometric stability may reflect a species-specific constraint or an adaptive priority to maintain a consistent mechanical design across heterogeneous environments, possibly related to wind firmness or light competition [26]. This result aligns with previous studies on other tree species that found allometric invariance despite large differences in site productivity [27]. Nevertheless, the slightly higher b value on plains (0.68) compared to hills (0.48) and low mountains (0.59), though not statistically significant with the current sample sizes, hints at a possible tendency toward greater relative height investment under more favorable conditions. Future studies with larger sample sizes and broader environmental gradients may detect more subtle allometric shifts.

## 5. Conclusion

This study quantified the growth responses of *Robinia pseudoacacia* plantations to landform, altitude, and slope in central Shandong. Trees on plains had significantly greater DBH and height than those on hills and low mountains. Both altitude and slope showed significant negative correlations with DBH and height: per 100 m increase in altitude, DBH decreased by 0.58 cm and height by 0.47 m; per 10° increase in slope, DBH decreased by 0.76 cm and height by 0.57 m. Altitude explained slightly more growth variation than slope, and DBH was more sensitive than height. The height–DBH allometric relationship did not differ significantly among landforms (ANCOVA, P = 0.776), with scaling exponents of 0.59 (low mountain), 0.48 (hill), and 0.68 (plain). These results provide empirical, correlative references for site evaluation and growth prediction in similar environments, but causal mechanisms remain to be tested with additional soil and climatic data.
